# Development of the reading literacy questionnaire for EFL learners at primary schools

**DOI:** 10.3389/fpsyg.2023.1154076

**Published:** 2023-05-12

**Authors:** Weilai Li, Shumin Kang, Yanhong Shao

**Affiliations:** ^1^Faculty of Education, Qufu Normal University, Qufu, Shandong, China; ^2^College of Foreign Languages, Qufu Normal University, Qufu, Shandong, China

**Keywords:** EFL learners, assessment instrument, reading literacy, elementary level, questionnaire development

## Abstract

Previous studies have indicated that there are a variety of factors influencing reading literacy assessment, including linguistic, cognitive, and affective factors, but little has been done on how to integrate these influential factors reasonably in a reading literacy instrument. As such, the purpose of this study is to develop and validate an English Reading Literacy Questionnaire (ERLQ) for English as foreign language (EFL) learners at the elementary level. The ERLQ was designed and revised through three rounds of validation with a sample of 784 pupils (Grades 3–6) in six primary schools from six provinces in China. Validity and reliability tests of the questionnaire were conducted with item analysis, Exploratory Factor Analysis (EFA), Confirmatory Factor Analysis (CFA), reliability test, and the analysis of criterion validity in SPSS 26.0 and AMOS 23.0. Results indicated that the revised ERLQ had high internal consistency, ranging from 0.729 to 0.823. The criterion validity of the ERLQ was supported by significant correlations to the Chinese Students’ English Rating Scale verified by the authoritative department, with a correlation coefficient of 0.871. The study shows that the revised questionnaire, with 3 dimensions of 14 items, has high reliability and validity, which can be used as an assessment instrument for the intended audience. It also suggests that modifications may be made for further use in other regions and countries, depending on the background information of the learners.

## Introduction

Reading is a crucial approach to language acquisition (Avalos et al., 2007; Goldenberg, 2020), which has a far-reaching influence on people’s learning and thinking (Green et al., 2013). The important role that it plays in students’ growth and academic development has made it a hot topic of research. At present, most of the research on reading have focused on the direct or indirect impact of linguistic, cognitive, and affective factors (Cheung et al., 2009; Nevo et al., 2020), even gender (Solheim and Lundetræ, 2018; Reilly et al., 2019) and parental literacy (Hunstinger et al., 2016) of learners, and the development of regional economy (Chinyamurindi and Dlaza, 2018). However, little has been done on how to integrate these influential factors reasonably in an instrument to assess students’ reading literacy, especially for teenagers in the context of English as a foreign language (EFL). As such, it is necessary to develop a self-report questionnaire to assess EFL learners’ reading literacy, especially at the elementary level, for they are at the vital stage of language development. The following two questions are formulated to guide the study. (1) What dimensions and factors should be considered in developing an instrument to assess EFL learners’ reading literacy at the elementary level? (2) What are the crucial steps in developing and validating the assessment instrument?

## Literature review

### The connotation of reading literacy

The concept of reading literacy is derived from the rethinking of the issue whether reading comprehension is viewed as a product or a process (Dyke, 2021). The former tends to highlight the content of what is read with sets of questions, whereas the latter pays much attention to the cognitive process in which how the influential factors contribute to the meaningful construction of the reading materials, concerning word identification, strategy use, and some cognitive behaviors (Broek et al., 2012). At present, the dominant and popular view of the connotation of reading literacy is in line with the latter that reading literacy is considered as a cognitive process involving multiple influencing factors such as linguistic knowledge and psychological behaviors. According to *the PISA* (Program for International Student Assessment) *2018 Reading Assessment Framework* by the Organization for Economic Co-operation and Development (OECD, 2019), reading literacy refers to understanding, using, evaluating, reflecting on, and engaging with reading materials or texts, which encompasses metacognitive, cognitive, and affective-motivational dimensions of behaviors. In *the PIRLS* (Progress in International Reading Literacy Study) *2021 Assessment Framework* by the International Association for the Evaluation of Educational Achievement (Mullis and Martin, 2019), the definition of reading literacy, emphasizes a constructive and interactive process between the reader and the author. Similarly, the National Assessment of Educational Progress of the US (NAEP) states that reading literacy is an active and complex process that involves understanding the written text, developing and interpreting its meaning from the context (U.S. Department of Education, 2022). Pan-Canadian Assessment Program (PCAP) also claims that reading literacy is the ability to construct meaning from texts through understanding, interpreting, and responding personally and critically to text content in order to make sense of the world based on personal life experience (O’Grady et al., 2021). All the above views denote that the connotation of reading literacy has gone beyond the reading activity itself, which is closely related to the development of the reader’s comprehensive reading competence.

Taken together, reading literacy in this study can be defined as a kind of integrated competence to achieve self-development through understanding and interpreting reading materials with evaluative and reflective abilities, within which language knowledge (e.g., morphology and syntax) is the basis for constructing the literal meaning of reading materials (Charles and Joseph, 2014). Readers with better reading literacy can adopt reading strategies and skills selectively to achieve different purposes of reading tasks (Svjetlana and Igor, 2006).

### The assessment of reading literacy

The assessment of reading literacy should not only focus on the static measurements of pre-existing reading competence, but also on dynamic assessment, which is an approach to psychological testing that conceptualizes the cognitive ability of readers (Dixon et al., 2023). To date, most of the international assessment institutions of reading literacy like PISA, PIRLS, and NAEP focus more on proficiency tests of reading (Rindermann, 2007; Applegate et al., 2009), whose purpose is to find out whether learners have already attained the knowledge and the skills in reading (Alderson and Hughes, 1981). Different from proficiency tests, self-report questionnaires rely on an individual’s own report of his or her behaviors, beliefs, or attitudes (Levin-Aspenson and Watson, 2018), which is commonly used in psychological studies because it can reveal the underlying causes of the concerned behaviors and yield some diagnostic information (Gollan et al., 2012). Specifically, reading literacy assessment concerns readers’ linguistic knowledge, reading strategies, even cognitive aspects in reading activities. Research revealed that the assessment of reading literacy involved complex interactive and dynamic processes (Sadeghi, 2021), not only including the readers’ linguistic and cognitive competences (NGA and CCSSO, 2010), but also containing the underlying impact of readers’ affective variable on reading (Adrian et al., 2007).

As a two-way interaction between the author and the reader (Cox et al., 2019), reading literacy is first affected by the linguistic competence of the reader, which is the most important element of reading literacy (García and Cain, 2014). According to OECD (2021), a reader’s linguistic competence refers to the ability to read effectively, largely depending on the comprehensive use of language knowledge and reading skills. Reader’s language competence necessitates a set of language knowledge and skills to derive textual meaning (Grabe, 2009). In this sense, linguistic competences such as word recognition, phonetic decoding, and syntactic parsing, can largely contribute to the construction of text meaning (Gellert and Elbro, 2017; Quintino and Julia, 2018), which should be taken into account in assessing reading literacy (NRP, 2000; Kirby et al., 2012; Li et al., 2012).

Cognitive competence, in the field of reading, refers to the ability to carry out an independent cognitive reading activity in a broad sense (Anikina, 2022), focusing on the process of turning knowledge into common sense, and then forming self-judgment on textual meaning (Sun and Hui, 2012). Previous studies (Rendell et al., 2010; Watkins, 2016) claim that cognitive strategy and cultural awareness pertain to different assessment facets of cognitive competence, which are considered as accelerators in facilitating the development of one’s reading literacy (Arabski and Wojtaszek, 2011; Piasta et al., 2018). Strategies used in reading activity comprises planning, monitoring, and mediating strategies that are embedded in reading behaviors, which have a potential impact on reading outcome (Aghaie and Zhang, 2012). Besides, cultural awareness, as a component of language proficiency, is a latent variable for better reading performance (Knutson, 2006; Byram, 2012; Baker, 2015), which contains the sensitivity to customs, traditions, values and beliefs of a specific community. Readers with different cultural backgrounds may have different comprehension and interpretation of the same reading materials. Cognitive strategy and cultural awareness jointly compose cognitive competence that has been a determinant component for the assessment of reading literacy.

As a new assessment dimension of reading literacy, affective element has become another important component in the sustainable development of one’s reading literacy, which consists of a series of factors, concerning reading attitude (Kaderavek et al., 2014; Nootens et al., 2018; Lopes et al., 2022), reading anxiety (Katzir et al., 2018), reading motivation (Schiefele et al., 2012; Stutz et al., 2017; Wang and Gan, 2021; Wang and Jin, 2021), and reading habits. Thus, in recent years, a new trend of research has been aroused, on how to assess learners’ affective impact on reading performance such as attitude and interest, showing the feature of diversified development of reading literacy assessment (Kell and Kell, 2014). Some researchers hold that students’ reading performance is closely related to their cognitive and affective factors (Chiu et al., 2012). Some think that affective factors have a potential influence on the reading proficiency of readers (Li, 2018). Others insist that individual differences should not be ignored in the process of reading literacy assessment (Conrad et al., 2013). Despite the proliferation of research on the affective effects of learners on reading performance, few studies have been made on what affective elements should be considered and integrated into reading assessment, and to what extent every influential element contributes to the development of reading literacy of the learner.

### Reading literacy assessment in EFL context

The effect of whether English is used as the mother tongue or a foreign language on English reading.

literacy assessment cannot be ignored (Zuikowski et al., 2019; Calet et al., 2020). Different language environment may have different impacts on the requirement of reading literacy assessment. As a foreign language, reading in English at primary schools belongs to the stage of “learning to read,” focusing on the basic knowledge of the target language and understanding the basic meaning of reading materials. The process of reading is naturally associated with the theme, structure of the reading materials, the interaction with different views, and the abilities of one’s open-mind thinking and psychological cognition (Education Bureau of the Hong Kong, 2022). Thus, in countries where English is a foreign language learnt formally in a non-native-speaking social context, the assessment of English reading literacy focuses more on its connection with English education, embodying the disciplinary characteristics of English learning. English reading literacy in the EFL context is usually thought of as four dimensions: language ability, cultural awareness, thinking quality, and learning ability, according to the Ministry of Education of the People’s Republic of China (PRC; Ministry of Education of the PRC, 2020; Ministry of Education of the PRC, 2022). All the dimensions are interrelated and supported to reflect the core value of the English course. In view of this, numerous factors such as thematic content, text structure, language knowledge, and non-language factors have been put forward in assessing students’ reading literacy (Wang, 2012; Liu and Wu, 2019). It is believed that reading comprehension ability is the result of the joint action of a series of reading behaviors of identification, analysis, and critical evaluation.

In sum, previous research on English reading literacy assessment presented different views, but few focus on the design and validation of English reading literacy assessment instruments, especially for primary school students in the context of a non-native-speaking social context, namely, learning English as a foreign language (EFL). As such, the current research is designed to develop an instrument for the assessment of English reading literacy for EFL learners, with an attempt to provide some empirical support for the study of English reading literacy.

## Questionnaire development

The development of English reading literacy questionnaire (ERLQ) is composed of two stages. The first stage aims to select the appropriate dimensions for assessing English reading literacy by referring to previous research findings and relevant official documents about reading requirements for EFL learners. The second stage is concerned with the development of specific assessment items through interviewing some experts, researchers and teachers in the field of English education. Specifically, the design of the English reading literacy assessment questionnaire is mainly based on the following two aspects: one is the core literacy requirement of English discipline, and the other is the requirement of English reading practice. The whole selection process of assessment dimensions and items referred to the in-depth analysis of relevant literature and *China’s Standards of English Language Ability* by the Ministry of Education of PRC, which is believed with high validity and reliability due to its strict verification from authoritative departments. With this solid foundation available, the assessment dimensions of English reading literacy are developed with the domains of language competence, cognitive strategy, and affective element. Then the questionnaire is designed in the form of a self-report, which can facilitate the collection of a large amount of quantitative data (Robin and Scott, 2015). In order to avoid invalid answers, the question items are presented in the form of declarative sentences with a 5-point Likert scale ranging from 1 (completely inconsistent) to 5 (fully consistent), which is easy to rate the answers and standardize the results. Considering the cognitive feature of the target group, short sentences with ordinary words are used to make sure that they can fully understand the meaning of the statements.

**Figure 1:**
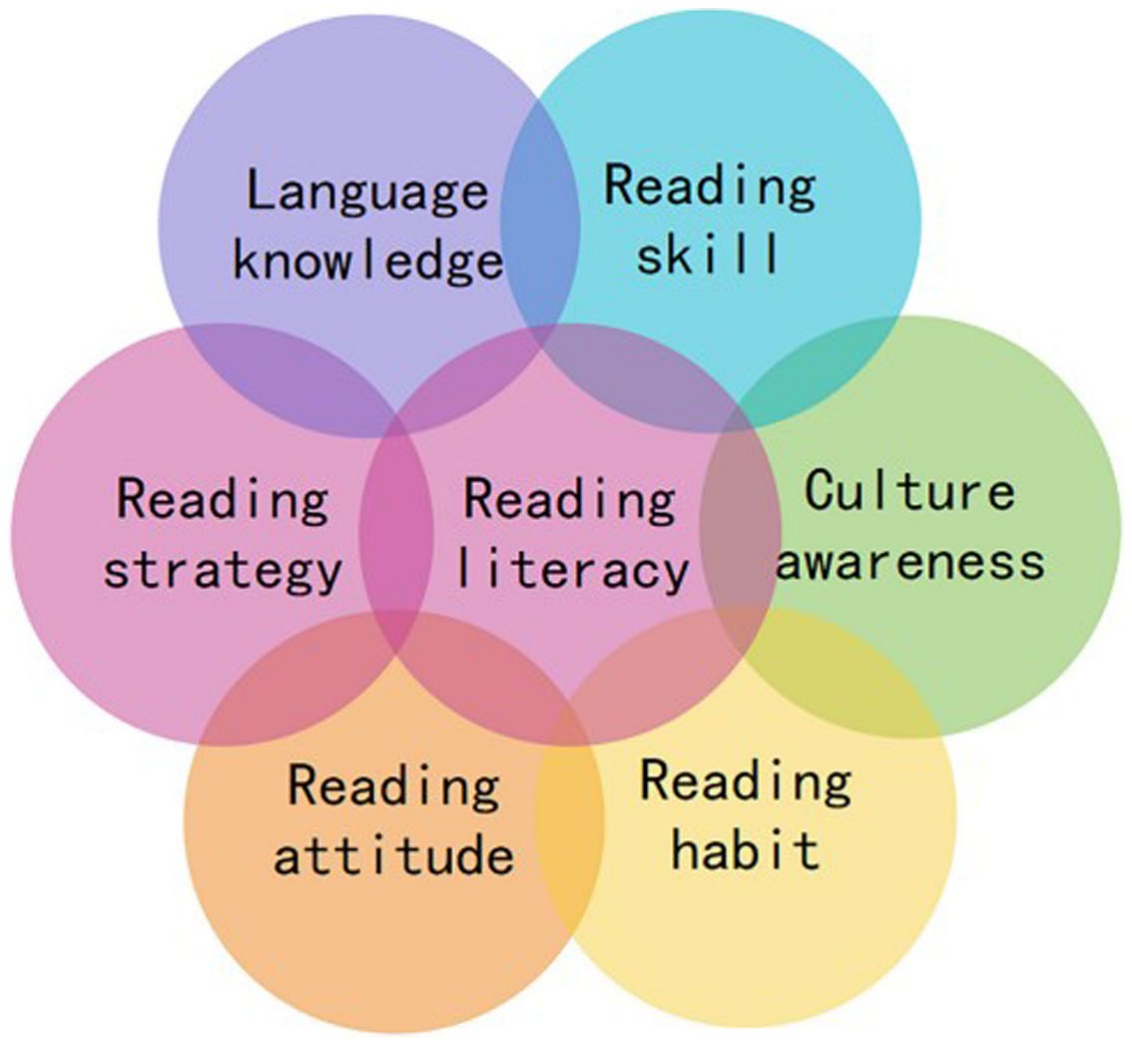
Six assessment dimensions of English reading literacy.

In order to make the questionnaire more specific and applicable, an interview was made for further suggestions, including 2 researchers on English teaching, and 10 English teachers. On this basis, the questionnaire items were tentatively selected and formulated. And the initial questionnaire is submitted to 3 English teaching experts from universities for further revision. Then, the assessment framework is constructed from 6 dimensions, namely: language knowledge, reading skills, reading strategies, cultural awareness, reading habits, and reading attitudes (see Figure 1) with 21 items (see Table 1).

**Table 1:** Assessment framework.

Number	Assessment item
Q1	I usually spend about 30 min reading English books every day.
Q2	I read English extracurricular books every day.
Q3	I often take notes while reading English books.
Q4	I’m willing to learn more through reading in English.
Q5	My reading can meet the basic requirements of the English course.
Q6	I can pronounce new words correctly.
Q7	I usually guess the meaning of new words from the context.
Q8	I can quickly grasp the main idea of English reading materials.
Q9	I can pronounce vowels and consonants of English words.
Q10	I can read new words based on the rules of alphabetic pronunciation.
Q11	I will not stop reading only because of the unknown words in the text.
Q12	I have mastered most of the English vocabulary required by the standard.
Q13	I have mastered the basic English grammar rules required by the standard.
Q14	I can read English books fluently.
Q15	I can get the information I want from reading materials.
Q16	I can use different reading methods as needed.
Q17	I can understand the meaning of text from different contexts.
Q18	I can communicate with others in simple English.
Q19	I know some customs of English-speaking countries.
Q20	I like reading books about the culture of my own country.
Q21	I can make a simple comparison between Eastern and Western cultures.

## Methods

### Sampling and data collection

The samples for this study were 784 primary school students (Grades 3–6) from six schools of six provinces representing different educational levels in China (2 in Eastern China, 2 in South China, and 2 in North China), within which 48.3% were boys (*N* = 379) and 51.7% were girls (*N* = 405). Data were collected via self-reported questionnaires analyzed with the software SPSS 26.0 and Amos 23.0. First, the consent to carry out the survey was obtained from the Research Ethics Committee, the headmasters of the participant schools, and the parents of the participants. Second, the content and purpose of the survey were explained to the teachers and students in detail. Third, the questionnaires were completed in class and the students were told that the participation was anonymous and voluntary, and encouraged to make their choices faithfully.

Specifically, data collection is composed of two stages. In the first stage, in February of 2022, 450 copies of the first version of the questionnaire were handed out and 420 valid questionnaires were obtained for data analysis, with an effective response rate of 93.3%. To obtain an appropriate sample size for factor analysis, we used a sample-to-item ratio of 10:1 (Hair et al., 2010). As the initial questionnaire contains 21 question items, the 420 samples were divided into two parts on average by grade: Sample Group A (*N* = 210) for predictive validation including item analysis, and exploratory factor analysis (EFA), within which 49.5% were boys (*N* = 104) and 50.5% were girls (*N* = 106); Sample Group B (*N* = 210) for construct validation including confirmatory factor analysis (CFA), within which 48.1% were boys (*N* = 101) and 51.9% were girls (*N* = 109). After a series of analyses, the first version of the questionnaire was revised with 3 dimensions of 14 items. In the second stage, in March of 2022, the revised questionnaire was handed out to the same six schools, together with Chinese Students’ English Rating Scale verified by authoritative departments, whose data were collected for the analysis of criterion validation. Each participant was asked to fill in both the questionnaire and the scale. Three hundred and sixty four valid questionnaires and 364 valid scales were obtained, with an effective response rate of 91%. Of the 364 participants, 47.8% (*N* = 174) were boys and 52.2% (*N* = 190) were girls.

### Data analysis

This study is conducted to develop and validate an English Reading Literacy Questionnaire for EFL learners at primary schools, following the established guidelines for test procedure made by American Educational Research Association, American Psychological Association, and National Council on Measurement in Education (2014). To determine whether the ERLQ has a good validity, a series of analyses were conducted, including content analysis, item analysis, exploratory factor analysis, confirmatory factor analysis, reliability analysis, and correlation analysis.

Specifically, the validation of the ERLQ involved three steps: (1) predictive validation. Predictive validation is composed of content analysis, item analysis, and Exploratory Factor Analysis (EFA), aiming to test the validation of questionnaire content, the distinction degree of the questionnaire items respectively, and to determine whether the factors of the questionnaire are reasonable for reading literary assessment. The predictive validation of the first version of the ERLQ was analyzed with the data of Sample Group A. (2) construct validation. Construct validity refers to the corresponding relationship between assessment dimensions and measurement variables, whose aim is to test whether the construct of the questionnaire is reasonable with the measurement methods of Confirmatory Factor Analysis (CFA; Schmitt, 2011). Six indices were used: Chi-squared divided by degrees of freedom (CMIN/DF), goodness-fit index (GFI), adjusted goodness-of-fit index (AGFI), normed fit index (NFI), incremental fit index (IFI), and root-mean-square error approximation (RMSEA). (3) criterion validation and reliability. The reliability of the revised questionnaire was tested for its internal consistency, which can reflect the extent to which the revised questionnaire measures English reading literacy (Livingston, 2018). Criterion validity is an index to test the quality of a questionnaire through the analysis of the relationship between the measured data and the criterion. The criterion scale was the Chinese Students’ English Rating Scale verified by authoritative departments with high validity and reliability. A correlation analysis between the revised questionnaire and the criterion scale was made by providing the value of the correlation coefficient. The criterion validation and reliability were tested with the survey data of 364 participants in the second stage of data collection.

## Results of predictive validation of the initial ERLQ

### Content validation

Content validation refers to the appropriateness of the questionnaire items to the measurement of relevant dimensions, and whether the items can reflect the connotation of the assessment dimensions or can appropriately measure the domain of the content under consideration. At the design stage of the ERLQ, we conducted an interview for the evaluative judgement on the content of the questionnaire, with experts in the field of English teaching, teachers from primary schools, and researchers from research institutions. All of them thought that the questionnaire contained representative items of the domain of reading literacy and that the item statements were suitable for EFL students at the elementary level. In view of this, the ERLQ can be considered with acceptable content validity.

### Item analysis

The scores of all items were analyzed for statistical significance by calculating the value of standard deviation. The method of convergence analysis was used with the valid data of Sample Group A. It is believed that if the standard deviation of each question item is below 0.5, it ought to be deleted for its convergence with other question items (Marjanovic et al., 2015). The results show that the standard deviation of all items is between 0.942 and 1.303, which meets the requirements of distinction degree; The correlation coefficient between the score of each item and the total score was statistically significant (0.591 ≤ *r* ≤ 0.852, *p* < 0.01). Then, the data were arranged in descending order by the scores. The first 27% was taken as the high group and the last 27% as the low group. Due to the normal distribution of the data, the independent sample T-test was used to test the discriminant validity (*p* < 0.05) and criteria value (*t* ≥ 3.00) of high and low groups on each item. The results show that the scores of all questions were statistically significant in the high and low groups (*p* < 0.01; see Table 2), indicating that the question items were suitable for further analysis.

**Table 2:** T-test of high and low group and correlation analysis between each item and the questionnaire.

Items	*M ± SD*	*t*	*r*	Items	*M ± SD*	*t*	*r*
1	4.04 ± 1.099	7.196	0.591^**^	12	3.78 ± 1.018	9.649	0.705^**^
2	3.97 ± 0.955	9.740	0.728^**^	13	3.79 ± 1.073	14.321	0.852^**^
3	3.98 ± 0.942	8.197	0.713^**^	14	3.84 ± 1.077	13.717	0.815^**^
4	3.77 ± 0.967	8.315	0.704^**^	15	4.03 ± 0.960	7.950	0.721^**^
5	3.60 ± 1.081	11.239	0.798^**^	16	3.82 ± 1.052	10.410	0.765^**^
6	3.29 ± 1.147	12.533	0.731^**^	17	3.84 ± 1.062	13.308	0.860^**^
7	3.64 ± 1.162	11.389	0.787^**^	18	3.96 ± 0.973	9.312	0.753^**^
8	3.95 ± 1.045	8.982	0.710^**^	19	3.79 ± 1.052	10.583	0.713^**^
9	3.60 ± 1.134	13.627	0.820^**^	20	3.66 ± 1.114	10.451	0.789^**^
10	3.60 ± 1.303	13.343	0.835^**^	21	3.90 ± 1.075	8.070	0.667^**^
11	3.71 ± 1.167	9.507	0.715^**^				

^**^p < 0.01.

### Exploratory factor analysis

The analysis of the exploratory factor was also performed for the load value of each factor, with the data of Sample Group A. First, the feasibility of factor analysis was carried out. Results show that most of the correlation coefficients in the correlation matrix are greater than 0.3; the value of Kaiser-Meyer-Olkin (KMO) is 0.902, greater than 0.7; The *value of p* of Bartlett sphericity test is 0, less than 0.05, indicating that it is suitable for exploratory factor analysis. Second, the principal component analysis method and the maximum variance method were adopted for factor rotation analysis. After multiple rounds of factor rotation test, three items, namely, Q1, Q10, and Q11 were deleted for their values of factor load were lower than 0.5. Four items (Q9, Q14, Q15, and Q17) were deleted because the content of these items was not so consistent with that of the other items in this dimension. The change of six dimensions to three dimensions is not a simple deletion, but rather an integration between dimensions because of their theoretical relations. For example, language knowledge is the basic condition for improving reading skills, which can in turn help learners acquire more language knowledge. The two dimensions are integrated into one dimension namely knowledge and skills, which is handy and valid for assessing learner’s reading literacy in this aspect. Finally, 7 question items were finally deleted and the 6 assessment dimensions were condensed into 3 dimensions, namely, habit and attitude, knowledge and skills, and culture and strategy. The remaining 14 items could better reflect the whole connotation of English reading literacy, whose cumulative contribution rate is up to 59.876% shown in Table 3, implying that the three assessment dimensions could basically reflect the level of learners’ reading literacy. After the analysis of factor rotation, the correlation of between items is high, and the load value of each item is between 0.536 and 0.825 (see Table 4), indicating that the questionnaire has good construct validity and content validity.

**Table 3:** Explained total variance.

Component	Initial Eigenvalue			Sum of squares of rotating loads		
	Total	Percentage variance	Accumulation %	Total	Percentage variance	Accumulation %
1	6.103	43.596	43.596	2.966	21.182	21.182
2	1.332	9.518	53.114	2.771	19.792	40.974
3	0.947	6.762	59.876	2.646	18.902	59.876

**Table 4:** Dimensions and assessment items.

Codes	Dimension	Assessment items	Load value
Q2	Habit and Attitude	I read English extracurricular books every day.	0.770
Q3		I often take notes while reading English books.	0.626
Q4		I’m willing to learn more through reading in English.	0.753
Q5		My reading can meet the basic requirements of the English course.	0.660
Q6	Knowledge and Skill	I can pronounce new words correctly.	0.752
Q7		I usually guess the meaning of new words from the context.	0.597
Q8		I can quickly grasp the main idea of English reading materials.	0.623
Q12		I have mastered most of the English vocabulary required by the standard.	0.760
Q13		I have mastered the basic English grammar rules required by the standard.	0.580
Q16	Culture and Strategy	I can use different reading methods as needed.	0.538
Q18		I am willing to share ideas from reading with others.	0.521
Q19		I know some customs of English-speaking countries.	0.758
Q20		I like reading books about the culture of my own country.	0.746
Q21		I am willing to make a simple comparison between Eastern and Western cultures.	0.827

## Results of construct validation of the revised ERLQ

**Figure 2:**
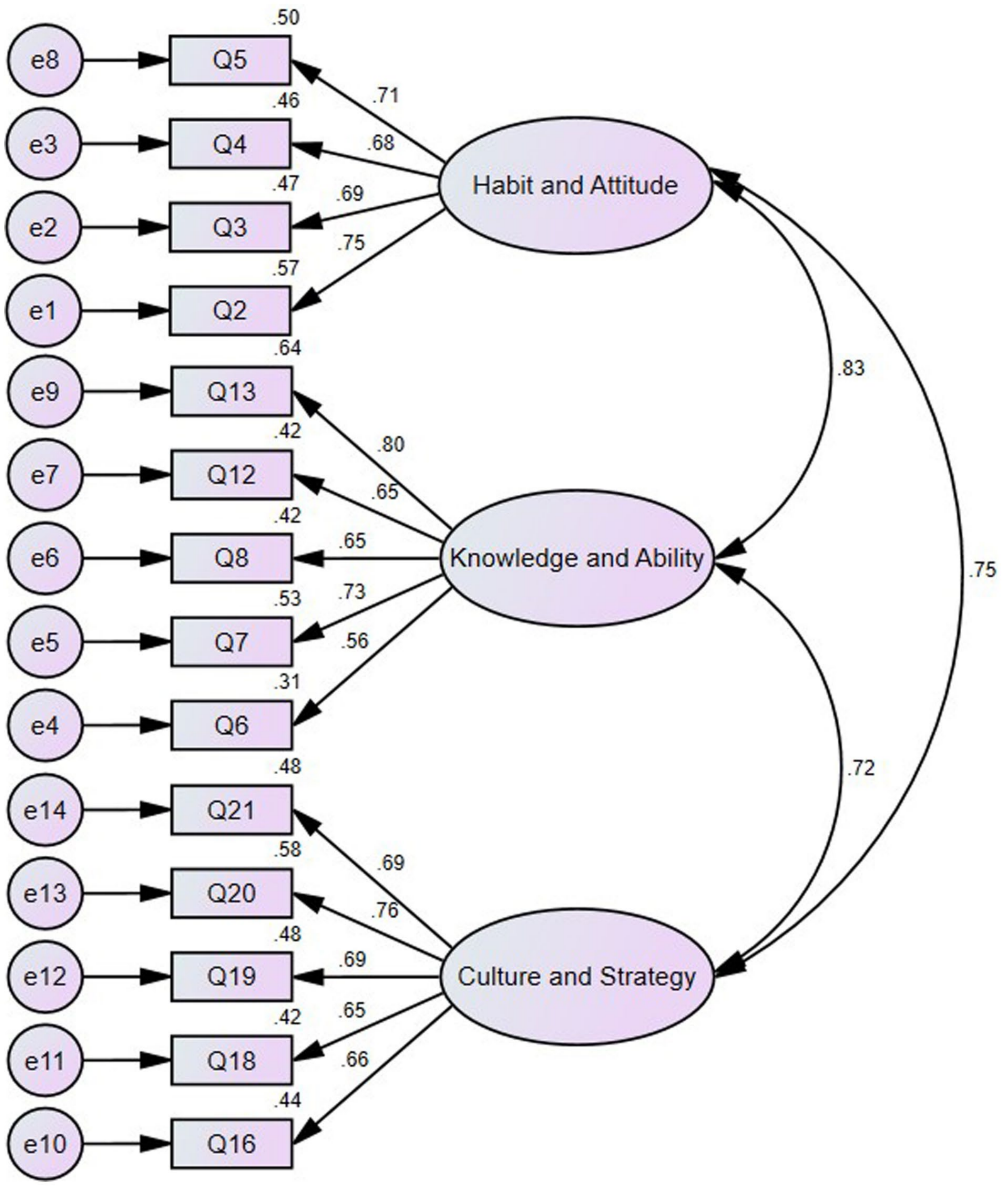
The improved structure of the questionnaire.

The construct validation of the revised ERLQ is tested with the data of Sample Group B. In the analysis of confirmatory factors with the data of Sample Group B, re-integration of dimensions was performed to test the fitting degree between the improved structure and new measured data. The Confirmatory Factor Analysis results are shown in Figure 2 that the factor load of each item is above 0.3, reflecting the factor load is relatively ideal as a whole. The correlation coefficient between the three dimensions is greater than 0.7, showing a strong positive correlation. The calculation results show that the value of CMIN/DF is 1.508, less than 3; GFI = 0.935, AGFI = 0.907, NFI = 0.909, IFI = 0.968, RMSEA = 0.049. According to various indicators, it is found that the structure of the questionnaire fits well with the measurement data, and the questionnaire has high construct validity.

## Results of reliability and criterion validation of the revised ERLQ

### Reliability

Reliability refers to the stability of a measurement instrument, which is usually tested by measuring its coefficient of Cronbach *α* (Livingston, 2018)f With 364 samples of survey data, this study used the software SPSS 26.0 to calculate the internal consistency coefficient of each dimension and the total questionnaire. The results show that the coefficient of Cronbach’s *α* of the ERLQ is 0.904, greater than 0.5 (Tabachnick et al., 2007), and the value of coefficient *α* of each dimension is between 0.729 and 0.823, which indicates that the internal consistency between each dimension and the total questionnaire is high and the ERLQ has high reliability (see Table 5).

**Table 5:** Reliability coefficient of the questionnaire.

Dimensions	Number of items	Cronbach’s *α* coefficient
Habit and attitude	4	0.729
Knowledge and skill	5	0.812
Culture and strategy	5	0.823
Total questionnaire	14	0.904

### Criterion validity

Criterion validity refers to the correlation of measurement results between a developed instrument and a criterion scale (Peterson et al., 2010). By calculating the total score of each student on all items, this study finally obtains two sets of score data based on the ERLQ and Chinese Students’ English Rating Scale, and conducts bivariate Pearson correlation analysis with the software of SPSS. The results show that the correlation coefficient is 0.871 (*p* < 0.01), implying a highly positive correlation between reading literacy and language proficiency (see Table 6), indicating that the improved assessment questionnaire has good criterion validity.

**Table 6:** Correlation analysis between the ERLQ and Chinese Students’ English Rating Scale.

		The ERLQ	Chinese students’ English rating scale
The ERLQ	Pearson correlation	1	0.871^**^
	Sig. (double-tailed)		0.000
	Number of cases	364	364
Chinese students’ English rating scale	Pearson correlation	0.871^**^	1
	Sig. (double-tailed)	0.000	
	Number of cases	364	364

** The correlation is significant at the level of 0.01 (double-tailed).

## Discussion

This paper presented the development of an assessment questionnaire for pupils on English reading literacy, during which, a variety of relevant test analyses were made for validation. Results show that the questionnaire developed in the current study has high validity and reliability, which can be used as an assessment tool for the intended EFL learners.

### The multidimensionality of reading literacy assessment

The results of the study showed that the 14 items extracted from the initial 21 items had sufficient load value to reflect the connotation of reading literacy. Specifically, the four-item dimension of habit and attitude, the five-item dimension of knowledge and ability, and the five-item dimension of culture and strategy exhibited a good level of fit in the structure of the ERLQ. The item-total correlation showed that all the items were proposed to be part of the ERLQ from moderately to strongly. All of these results further confirmed the multidimensionality of reading literacy assessment as suggested by previous studies (Fletcher, 2006; Lordán et al., 2017), demonstrating that reading literacy of EFL learners is affected by different purposes or reasons and that they have multifaceted differences in language, cognition and emotion as readers. This is consistent with the views of Scott (2011), who notes that language knowledge and skills are fundamental to reading, culture and strategy for deep comprehension of reading materials, habit and attitude for reading and lifelong development. The assessment of reading literacy can be understood as a multidimensional construction of different components related to the reading process, which is in line with previous findings (Mullis and Martin, 2019; OECD, 2019; O’Grady et al., 2021) that highlights the two-way interaction between the reader and the text. Despite the fact that there are many other dimensions and factors influencing reading literacy such as home literacy environment (Chow et al., 2015; Bergen et al., 2017; Zhang et al., 2020) and educational policy (Fuchs et al., 2019), it does not mean that the assessment dimension of a questionnaire is inclusive. The multidimensionality of reading literacy assessment has its limitation, especially for pupils, because the cognitive level of primary school students is still at the development stage, whose attention, memory, thinking, and other factors are in the transitional period from low-level to high-level development. The content and ways of assessment of English reading literacy naturally need to be in line with the cognitive characteristics of their ages.

### The correlation between students’ reading literacy and their language proficiency

The result of this study also showed that there was a strong positive relationship between reading literacy and language proficiency, with a value of the correlation of 0.871 gained from the criterion analysis, which is in accordance with previous studies (Stephen et al., 2002; Welcome and Meza, 2019), implying that language proficiency plays a crucial role in students’ reading literacy. The ERLQ’s reliability has been bolstered by its positive correlation with language proficiency. The correlation values in Figure 2 (e.g., 0.72, 0.75, and 0.83) indicate that the different dimensions are three distinct but highly interrelated constructs, indicating that reading literacy and language proficiency interact to influence reading performance. These findings provided empirical support to the construction of reading literacy assessment dimensions, which are in line with the views of Mirza et al. (2016) and Sun et al. (2022) who emphasize that excellent reading literacy is beneficial to the improvement of language proficiency, and vice versa. As such, in the EFL context, it is necessary to consider the effects that language proficiency has on reading literacy. We believe that sufficient language proficiency can effectively control the low-level ineffective reading process. Similarly, reading strategies can help readers comprehend the reading materials deeply. The correlations between students’ reading literacy and language proficiency indirectly illustrated the complexity of the reading process (Uccelli et al., 2015). To some extent, reading is a process of interaction, communication, and re-creation in thinking between the reader and author, requiring the integration and coordination of linguistic, cognitive, and affective factors. Notably, since reading literacy of a reader’s native language has positive transfer effect on that of his or her foreign language (Eibensteiner, 2023), reading habits and reading attitude matter more in the assessment framework than language proficiency (seen in Table 3), which is consistent with previous research (Kaderavek et al., 2014; Nootens et al., 2018; Lopes et al., 2022), demonstrating that individual behaviors of the reader should be measured more widely and deeply.

### The dynamic adjustment of ERLQ

The result of this study also indicated that the design of reading literacy assessment was a dynamic process, showing the features of openness, diversity, and constructiveness. In the exploratory factor analysis, 7 items were deleted from the first version of the questionnaire, further proving that it is necessary to choose appropriate aspects and relevant items for the assessment of reading literacy. It is very important to keep a balance between assessment standards and questionnaire design, in which the most credible aspects of reading literacy can be assessed. This finding is in line with previous research (Conrad et al., 2013; Sadeghi, 2021), highlighting the development of the reader’s psychological and cognitive behaviors in the reading process. Notably, reading literacy assessment itself is not a product, but a process, just like the well-known international reading literacy assessments, PISA and PIRLS, which release a new assessment framework every few years and improve the definition of reading literacy (Zuckerman et al., 2013). Different from previous findings that a standardized system was needed to assess English reading literacy, this self-report questionnaire can be adjusted dynamically to different research requirements with simple addition or modification of few items. With regard to the dynamic assessment of English reading literacy in EFL context, the key point is to reduce cultural and linguistic bias and focus on reading behaviors, which is consistent with previous researches (Petersen and Gillam, 2013; Navarro and Lara, 2017). Hence, the structure and content of the English reading literacy assessment questionnaire should be in an ongoing process of modification with the change of the target participants and the changing view of the reading concept. In terms of the assessment itself, it is not an end, but a new start to improve learners’ reading literacy.

## Conclusion

The development of the English reading assessment questionnaire is a multidimensional and dynamic process of component construction (Fletcher, 2006), not only involving linguistic and cognitive factors, but also affective elements such as attitude, habit, and motivation (Schiefele et al., 2012; Pekrun et al., 2017; Nootens et al., 2018), associated with the characteristics of the reading process and the research findings of reading literacy, together with the consideration of different learning stages and cultural background of the learner (Zuikowski et al., 2019; Calet et al., 2020). With the development of the English reading literacy assessment questionnaire in this study, some conclusions were made as follows: (1) The design of an assessment instrument for EFL reading literacy is a complex task, in which several factors should be fully considered, including the intended readers’ foreign language proficiency at different stages of learning and their cognitive and affective elements. (2) In the design process, relevant research results and different views about reading and reading literacy should be used for reference. In addition, different cultural and educational backgrounds, as well as different requirements or assessment standards, should be taken into consideration so as to meet the needs of assessment in specific regions or countries. (3) In terms of assessment, the developed questionnaire on English reading literacy in this study for students at primary schools has good reliability and validity, which can be used as an assessment tool for the intended audience.

It should also be noted that English reading literacy is an ever-developing concept. So is the development of its assessment. With the innovation of reading media and the change of reading methods, the connotation and extension of reading literacy will be enriched accordingly (Gil et al., 2015; Serafini et al., 2020). The assessment of reading literacy should also be updated. Although the cultivation of English reading literacy is a step-by-step process, it does not follow the development track from a single dimension to multi-dimension. It is a continuous process of accumulation of different dimensions.

### Implication and limitation

The study can make both theoretical and practical implications. Theoretically, on one hand, this study offers extended knowledge in constructing the assessment dimensions of reading literacy in the EFL context, which is conducive to the improvement of language assessment theory. On the other hand, as can be seen from the results, more dimensions and factors are likely to be included in future research, showing the diversified development trend of reading literacy. Practically, the developed questionnaire of this study may present a reading assessment model for educational practitioners in other EFL countries and regions. It is implied that the features of participants and places of residence should be fully considered in the design and application of any questionnaire.

Although the findings of this study have provided data-based support for the validity and reliability of the developed questionnaire, limitations should be acknowledged. The test sample is relatively small, and the participants are mainly from grade 3 to grade 6 in six primary schools in China. More participants need to be involved to validate the ERLQ for more convincing results. Besides, although the research sample covers most of the English learners of primary schools participating in this survey, this study did not make a distinction between the lower grade and the upper grade of the participants. Further research may be made to assess the differences between them.

It is hard to make an effective and universal assessment criterion appropriate to all EFL learners in different regions or countries. Thus, in the future, minor modifications of the ERLQ may be made for similar target audience in order to better meet different research purposes or requirements.

## Data availability statement

The raw data supporting the conclusions of this article will be made available by the authors, without undue reservation.

## Ethics statement

The studies involving human participants were reviewed and approved by the Research Ethics Committee of Qufu Normal University, the headmaster of the participant school, and the parents of the participants. Written informed consent to participate in this study was provided by the participants’ legal guardian/next of kin.

## Author contributions

WL organized the database, performed the statistical analysis, and wrote the paper. SK and YS contributed to conception and design of the study. All authors contributed to the article and approved the submitted version.

## Funding

This research was funded by the grant from the National Social Science Fund of China (grant number: BEA 180110).

## Conflict of interest

The authors declare that the research was conducted in the absence of any commercial or financial relationships that could be construed as a potential conflict of interest.

## Publisher’s note

All claims expressed in this article are solely those of the authors and do not necessarily represent those of their affiliated organizations, or those of the publisher, the editors and the reviewers. Any product that may be evaluated in this article, or claim that may be made by its manufacturer, is not guaranteed or endorsed by the publisher.

## Author disclaimer

The views expressed in this paper reflect the opinions of the authors and not the funding agency or the authors’ respective institutions.
